# Astragaloside IV mitigated cadmium-induced glandular gastric injury in ducks by inhibiting PANoptosis

**DOI:** 10.3389/fvets.2026.1926940

**Published:** 2026-08-18

**Authors:** Xuefeng Gao, Xueyuan Hu, Xilong Hao, Peng Zhao, Wenxuan Dong, Ji Sun, Zhixin Li, Xuefeng Lei, Guicheng Dong, Fu Chen

**Affiliations:** 1 College of Life Sciences, Inner Mongolia Agricultural University, Hohhot, Inner Mongolia, China; 2 Institute of Animal Nutritional Metabolic and Poisoning Diseases, College of Veterinary Medicine, Qingdao Agricultural University, Qingdao, Shandong Province, China; 3 College of Horticulture and Plant Protection, Inner Mongolia Agricultural University, Hohhot, Inner Mongolia, China; 4 College of Ecology and Environment, Baotou Teachers' College, Inner Mongolia University of Science and Technology, Baotou, Inner Mongolia, China; 5 Sinochem Modern Agriculture (Inner Mongolia) Co., Ltd., Hulunbuir, Inner Mongolia, China; 6 Baotou Institute of Agriculture and Animal Husbandry Science, Baotou, Inner Mongolia, China; 7 Agricultural College, Inner Mongolia Agricultural University, Hohhot, Inner Mongolia, China

**Keywords:** astragaloside IV, cadmium, duck, glandular gastric injury, PANoptosis, Sirt3

## Abstract

**Background:**

Cadmium (Cd), a nonessential heavy metal, threatens poultry health through environmental bioaccumulation; however, its adverse effects on the glandular stomach (GS), a critical digestive and immune organ in waterfowl, remain understood. The present study was designed to assess the gastroprotective potential of astragaloside IV (AS-IV), a naturally occurring triterpenoid saponin from *Astragalus membranaceus* with known anti-inflammatory and antioxidant properties, against Cd-induced glandular gastric injury in ducks.

**Methods:**

Ducks received long-term dietary AS-IV pretreatment prior to Cd challenge, and GS pathological, biochemical, and molecular analyses were subsequently performed.

**Results:**

AS-IV pretreatment significantly ameliorated Cd-induced pathological lesions, including glandular tubular architecture disruption, epithelial cell swelling, vacuolar degeneration, basement membrane sloughing, and mucosal necrosis, while reducing the GS index. Biochemically, AS-IV restored antioxidant enzyme activities, reduced lipid peroxidation, and downregulated pro-inflammatory cytokine production. At the molecular level, AS-IV downregulated the expression of pivotal biomarkers associated with apoptosis, pyroptosis, and necroptosis in GS cells. Bioinformatic analysis of protein–protein interactions additionally indicated plausible links between Sirt3 signaling cascades and PANoptosis-related mechanisms.

**Conclusion:**

Collectively, these results reveal that prophylactic dietary supplementation with AS-IV confers protective efficacy against cadmium-triggered glandular gastric damage in ducks, conceivably via the regulation of PANoptosis-associated pathways mediated by Sirt3. Such evidence underscores the potential of botanical extracts as a viable strategy for safeguarding gastrointestinal integrity in waterfowl exposed to heavy metal-induced toxicity.

## 1 Introduction

Cadmium (Cd), a highly toxic environmental pollutant capable of bioaccumulation and inducing hepatic, renal, and skeletal toxicity, is released into the environment mainly through industrial emissions, mining activities, and phosphate fertilizers (1). As Cd is not biodegradable, it readily accumulates in soils and water, then enters the food chain (2). Cd preferentially accumulates in the kidney, liver, bone, and brain, causing nephrotoxicity, hepatotoxicity, bone metabolic disorders, and neurodegenerative diseases (2, 3). Owing to a rapid growth rate and high feed consumption, ducks are highly susceptible to Cd toxicity (4). While the toxic mechanisms of Cd have been extensively studied in multiple organs of ducks (5–7), adverse effects on the gastrointestinal tract, particularly the glandular stomach (GS), remain underexplored. As a critical digestive and immune organ in poultry, dysfunction of the GS can severely impair nutrient absorption and overall health. However, to date, no study has yet investigated Cd-induced injury to the GS or the underlying molecular mechanisms, representing a significant gap in understanding the full spectrum of Cd toxicity in waterfowl.

Natural plant extracts with multi-targeting potential and bioactive properties have recently gained significant research interest. AS-IV is a naturally occurring triterpenoid saponin isolated from the root of *Astragalus membranaceus* (Fisch.) Bunge with anti-inflammatory, anti-apoptotic, antioxidant, and immunomodulatory activities (8). Previous studies have shown that AS-IV attenuated lipopolysaccharide-induced inflammatory injury in multiple organs, including the kidney, liver, and lung, by suppressing oxidative stress and inflammatory responses (9). Within this framework, parameters associated with oxidative stress, including malondialdehyde concentrations and superoxide dismutase (SOD) activities, are frequently regarded as composite indices reflecting overall physiological perturbations rather than discrete mechanistic pathways. In toxicological investigations employing avian models, these biomarkers have been extensively utilized to mirror the aggregate consequences of inflammatory responses, metabolic perturbations, and tissue damage (5). Taken together, these observations indicate that the anti-inflammatory and antioxidant capacities of AS-IV may confer organ protection across diverse experimental contexts. While hepatoprotective and nephrotective activities of AS-IV have previously been documented in select experimental systems (10), the potential to alleviate gastrointestinal injury, particularly glandular gastric injury induced by heavy metal exposure in ducks, remains insufficiently explored.

From the perspective of cell death regulation, emerging evidence indicates that AS-IV modulates programmed cell death pathways (11). PANoptosis, a recently identified form of inflammatory programmed cell death involving the coordinated activation of pyroptosis, apoptosis, and necroptosis, has been implicated in the pathogenesis of various toxicant-induced tissue injuries (12). Recent studies have demonstrated that Cd exposure triggers PANoptosis in multiple organs, contributing to tissue damage and functional impairment (13–15). Recent studies suggest that SIRT3, the principal mitochondrial NAD^+^-dependent deacetylase, functions as a crucial upstream regulator in renal injury pathogenesis and mediates crosstalk with PANoptosis (16). By deacetylating key metabolic and antioxidant enzymes, SIRT3 maintains mitochondrial homeostasis and prevents excessive ROS production. When SIRT3 function is compromised, mitochondrial ROS overproduction ensues, which simultaneously activates multiple programmed cell death pathways, including the RIPK1/RIPK3/MLKL necroptosis axis and cytochrome c-mediated apoptosis (17). Although the regulatory effects of AS-IV on individual cell death pathways have been progressively elucidated (8, 11), it remains uncertain whether AS-IV can protect against Cd-induced glandular gastric injury by activating SIRT3-mediated mitochondrial antioxidant defense and thereby suppress the PANoptosis pathway.

Accordingly, this study aimed to determine whether AS-IV administration could attenuate cadmium-induced glandular gastric damage in ducks, and whether its mechanistic basis involves suppression of oxidative stress, inflammation, and PANoptosis. A duck model of Cd-induced gastric injury was therefore established to evaluate the effects of AS-IV on gastric morphology and function, redox status, inflammatory responses, and regulated cell death modalities. Additionally, we sought to clarify whether the gastroprotective efficacy of AS-IV is linked to PANoptosis inhibition, thereby providing a theoretical basis for using botanical extracts to safeguard gastrointestinal integrity in waterfowl against heavy metal toxicity.

## 2 Materials and methods

### 2.1 Animal care and experimental design

All animal procedures were approved by the Institutional Animal Care and Use Committee of Baotou Teachers’ College, Inner Mongolia University of Science and Technology (approval no. AEWC-BTTCSTXY 2026-012), and performed in accordance with the Guide for the Care and Use of Agricultural Animals in Research and Teaching. Male Cherry Valley ducklings were randomly allocated to four groups (*n* = 30 each): control, Cd, AS-IV, and AS-IV + Cd. All ducklings received a basal diet formulated to NRC (1994) standards, with Cd provided as CdCl_2_. Following a 2-day acclimatization, the AS-IV and AS-IV + Cd groups were pretreated with AS-IV (80 mg/kg BW; purity ≥ 99%; MedChemExpress, Monmouth Junction, NJ, USA) by daily intragastric gavage for 28 days prior to Cd challenge. Meanwhile, the control and Cd groups received an equal volume of normal saline (0.9% NaCl) by daily intragastric gavage as a vehicle control. Concurrently, the Cd and AS-IV + Cd groups received diets supplemented with 25 mg/kg CdCl₂ throughout the study to mimic chronic exposure, whereas the control and AS-IV groups were maintained on Cd-free diets. Dosages were established by integrating effective ranges reported in prior animal studies with our own preliminary experiments to ensure both efficacy and safety (9). On day 28, blood was drawn from the jugular vein 4 h after the final feeding. The animals were then deeply anesthetized via intraperitoneal injection of sodium pentobarbital (80 mg/kg BW; Sigma-Aldrich, St. Louis, MO, USA) and humanely euthanized by cervical dislocation under deep anesthesia. GS tissues were immediately excised, rinsed with ice-cold saline, and preserved at −80 °C for further analysis.

### 2.2 Histopathological observation of GS tissues

For histological evaluation, GS tissues were fixed in 4% paraformaldehyde, routinely processed through ethanol dehydration, xylene clearing, and paraffin embedding, and sectioned at 4–5 μm. Slices were H&E-stained and examined under a light microscope.

### 2.3 GS index

After euthanasia, the GS was completely excised, trimmed of surrounding adipose and connective tissues, rinsed with pre-cooled physiological saline, and blotted dry. The wet weight was immediately recorded, and the GS index (%) was calculated as (GS weight/BW) × 100% (18).

### 2.4 Biochemical and inflammatory parameter analysis

GS homogenates were prepared by centrifugation (4 °C, 5,000×*g*, 10 min), and supernatants were collected for downstream assays. All biochemical parameters and inflammatory cytokines were subsequently quantified using commercial kits according to the manufacturer’s instructions.

### 2.5 Transmission Electron microscopy (TEM)

GS tissues were diced into 1-mm^3^ cubes and fixed in 2.5% glutaraldehyde in PBS (pH 7.4) at 4 °C for 24 h, followed by post-fixation in 1% osmium tetroxide. After dehydration through graded ethanol and acetone, specimens were embedded in epoxy resin and sectioned ultrathin. Sections were stained with uranyl acetate and lead citrate, then examined by TEM (HT7700; Hitachi, Tokyo, Japan) to assess ultrastructural alterations in GS cells.

### 2.6 Western blot analysis

Western blotting was performed as previously described (19), with protein concentrations determined by BCA assay (Solarbio, Beijing, China), and immunoreactive bands detected by ECL and quantified using ImageJ software.

### 2.7 Protein–protein interaction (PPI) network analysis

A PPI network between PANoptosis-related proteins and Sirt3 was constructed using the STRING database^1^ with a minimum interaction confidence score of 0.4, and the resulting network was visualized and exported as previously described (20).

### 2.8 Statistical analysis

Statistical analyses were conducted in IBM SPSS Statistics 27.0, with data visualization performed in Prism 9.5. Results are expressed as mean ± SEM. Normality and homogeneity of variance were examined by the Shapiro–Wilk and Levene’s tests, respectively. Intergroup comparisons were analyzed using one-way ANOVA followed by SNK *post hoc* test. Significance was set at *p < 0.05 or **p < 0.01.

## 3 Results

### 3.1 AS-IV attenuated cd-induced glandular gastric injury in ducks

**Figure 1:**
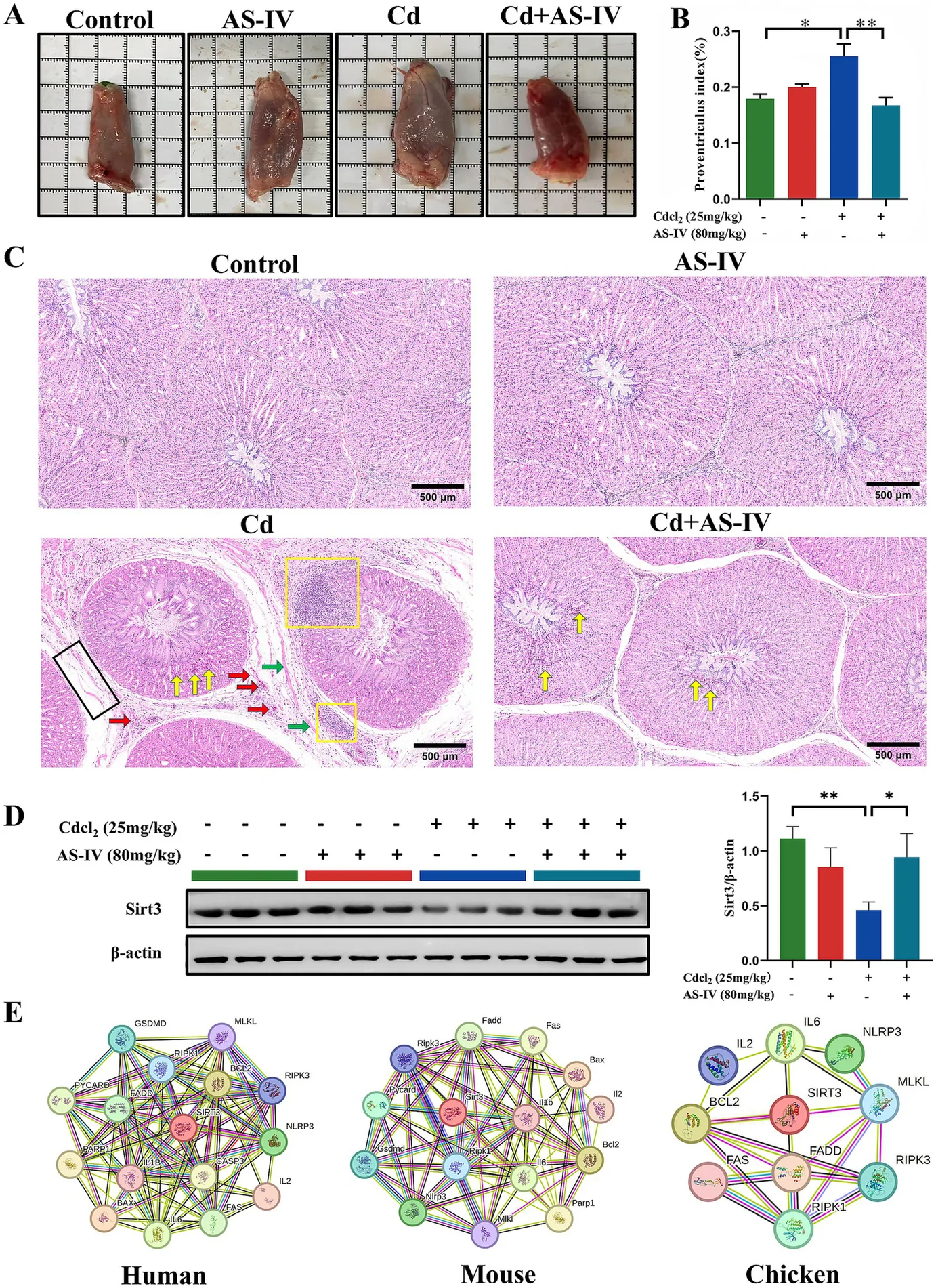
AS-IV attenuated Cd-induced glandular gastric injury in ducks. **(A)** Morphological changes to proventriculus tissue. **(B)** Proventricular index. **(C)** Representative H&E-stained proventriculus sections from the Control, Cd, AS-IV, and AS-IV + Cd groups (scale bar: 500 μm). Yellow arrows: Punctate and small focal necrosis of glandular cells. Red arrows: Interlobular connective tissue hyperplasia with congestion. Green arrows: Hyperplasia of mucosal muscular fibers. Black box: Severe edema. Yellow box: Lymphoid tissue hyperplasia of the mucosa. **(D)** Western blots of Sirt1 and *β*-actin levels in proventriculus tissue; the ratio of Sirt3/β-actin levels. **(E)** PPI network depicting relationships of Sirt3 with markers of necroptosis, apoptosis, and pyroptosis, and inflammatory cytokines. Data are presented as mean ± SEM (*n* = 3). *p < 0.05, **p < 0.01; ns, not significant.

As illustrated in Figures 1A,B, AS-IV intervention markedly alleviated Cd-induced enlargement of the GS and resulted in a lower GS index as compared to the Cd group. AS-IV administration markedly improved Cd-induced GS histopathological damage, as shown by intact glandular tubules, reduced epithelial injury, and attenuated interstitial inflammation (Figure 1C). Notably, severe mucosal layer necrosis and lymphoid tissue hyperplasia observed in the Cd group were markedly ameliorated following AS-IV administration. Western blot analysis showed that Cd exposure significantly suppressed Sirt3 protein expression in the GS, whereas AS-IV treatment effectively restored Sirt3 expression to near control levels (Figure 1D). Due to the absence of duck-related protein data in current PPI databases, cross-species network analyses were conducted using human, mouse, and chicken orthologs, all of which consistently placed Sirt3 within an interactive framework involving necroptosis, apoptosis, pyroptosis, and inflammatory mediators, thereby generating testable hypotheses regarding the mode of action (Figure 1E).

### 3.2 AS-IV alleviated cd-induced oxidative and inflammatory damage in the GS

**Figure 2:**
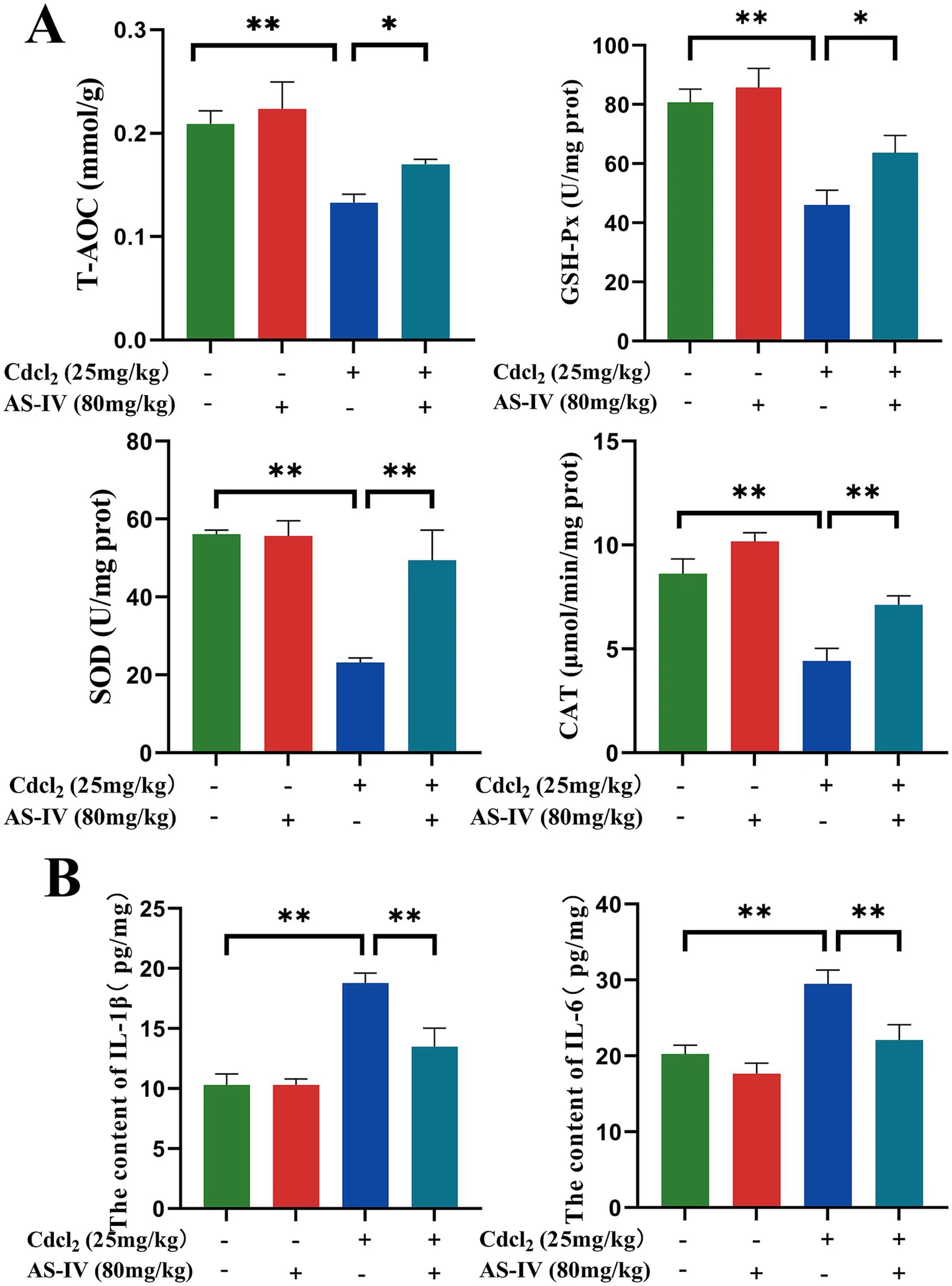
AS-IV alleviated Cd-induced oxidative stress and inflammatory responses in the duck GS. **(A)** The concentrations of T-AOC, GSH-Px, SOD and CAT in the proventriculus. **(B)** ELISA results of IL-1β and IL-6 levels in the proventriculus. Data are presented as mean ± SEM (*n* = 3). *p < 0.05, **p < 0.01; ns, not significant.

As depicted in Figure 2A, Cd exposure significantly disrupted the antioxidant defense system in the GS, as evidenced by markedly decreased activities of the indicators SOD, GSH-Px, CAT, and T-AOC as compared to the Control group. Notably, AS-IV intervention effectively restored the activities of these antioxidant enzymes, indicating attenuation of Cd-induced oxidative damage. Furthermore, the ELISA results revealed that Cd exposure markedly upregulated expression levels of IL-1β and IL-6 in GS tissues (Figure 2B). In contrast, AS-IV treatment suppressed the production of these inflammatory mediators, suggesting mitigation of Cd-induced inflammatory responses. Collectively, these results demonstrate that AS-IV protected against Cd-induced glandular gastric injury, at least partially, through amelioration of oxidative stress and inflammation.

### 3.3 AS-IV alleviated cd-induced apoptosis of duck GS cells

**Figure 3:**
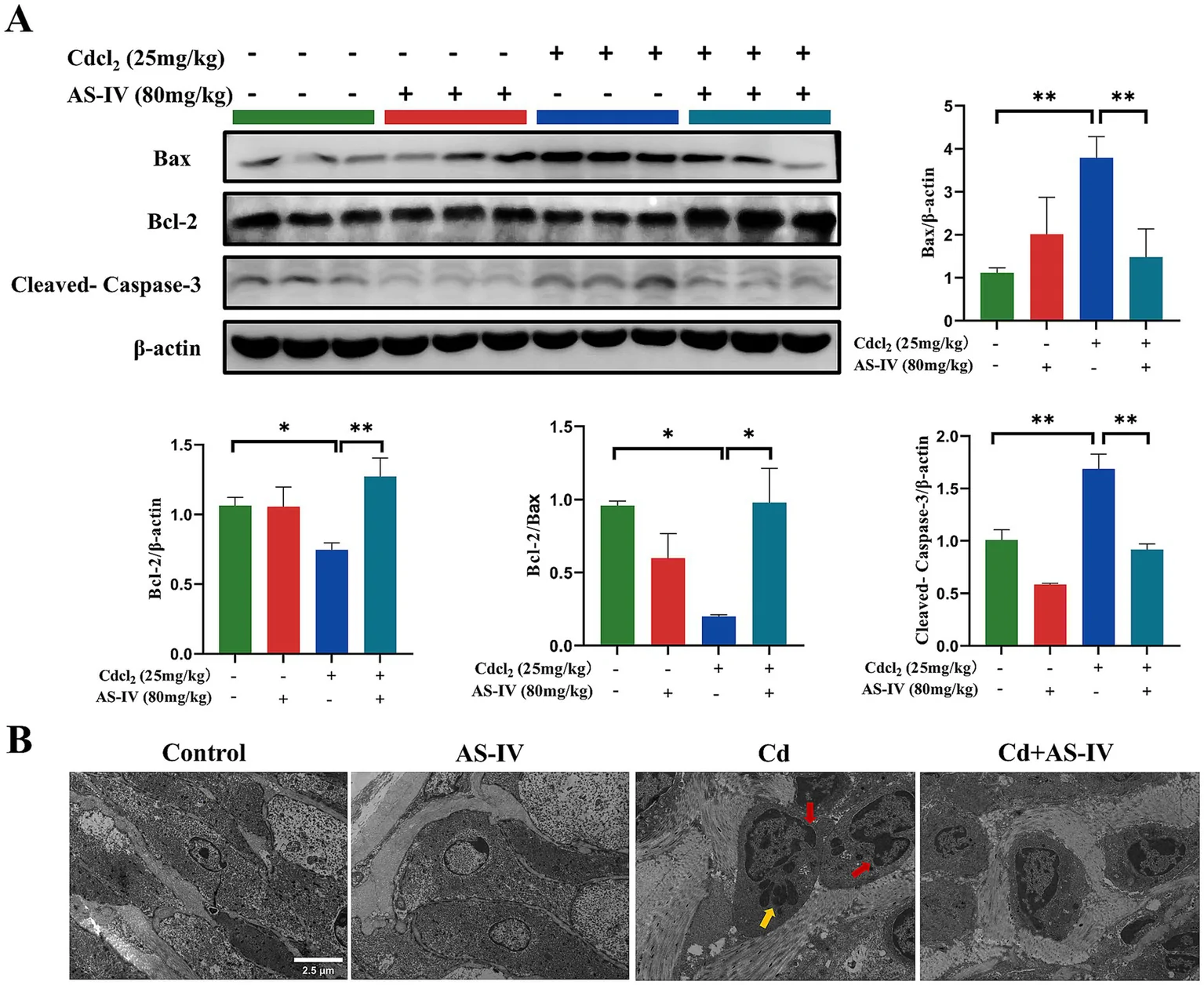
AS-IV alleviated Cd-induced apoptosis of duck GS cells. **(A)** Western blot results of Bax, Bcl-2, Cleaved-Caspase-3, and β-actin levels in proventriculus tissue; the ratios of Bax/β-actin, Bcl-2/β-actin and Cleaved-Caspase-3/β-actin levels. **(B)** Representative TEM images of the proventriculus. Red arrows indicate chromatin condensation; yellow arrows indicate mitochondrial swelling. Scale bar = 2 μm. Data are presented as mean ± SEM (*n* = 3). *p < 0.05, **p < 0.01; ns, not significant.

Western blot analysis revealed that Cd exposure significantly downregulated Bcl-2 and upregulated Bax, resulting in a marked decrease to the Bcl-2/Bax ratio as compared to the Control group (Figure 3A). Concurrently, expression of cleaved Caspase-3 was significantly elevated in the Cd group. TEM observation further corroborated these findings at the ultrastructural level (Figure 3B). The Control group exhibited intact cellular architecture with normal nuclear morphology and well-preserved mitochondrial cristae. By contrast, Cd-treated GS cells manifested classic apoptotic phenotypes, including nuclear chromatin clumping, nuclear disintegration, and apoptotic vesicle generation. Additionally, mitochondrial swelling with disrupted cristae and cytoplasmic vacuolization were evident in the Cd group. Importantly, AS-IV treatment markedly ameliorated these ultrastructural abnormalities, as the nuclear envelopes were relatively intact, chromatin was evenly distributed, and mitochondrial integrity was preserved.

### 3.4 AS-IV alleviated Cd-induced pyroptosis of duck GS cells

**Figure 4:**
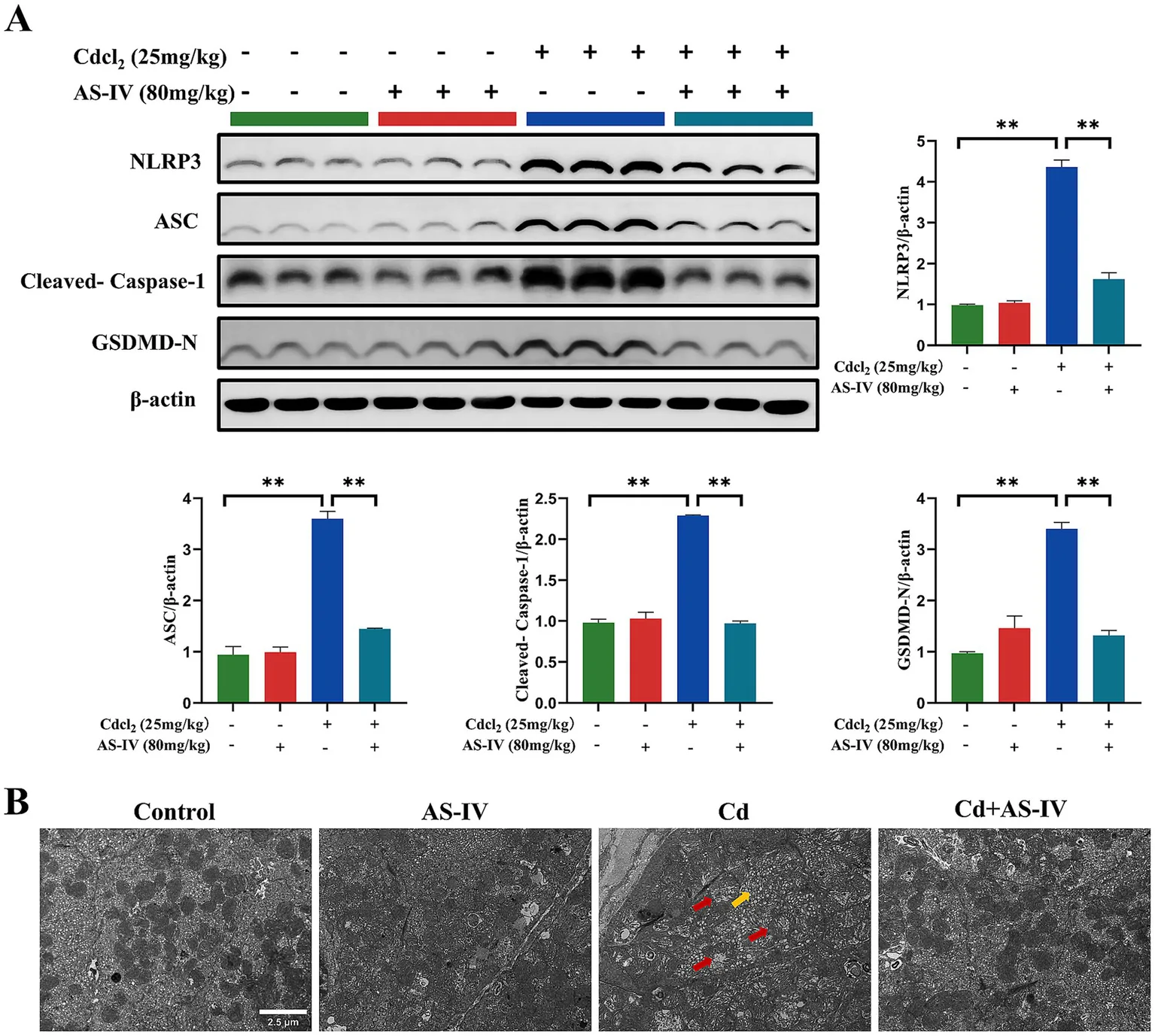
AS-IV alleviated Cd-induced pyroptosis of duck GS cells. **(A)** Western blot results of NLRP3, ASC, Cleaved-Caspase-1, Gasdermin D-N, and β-actin levels in proventriculus tissue; the ratios of NLRP3/β-actin, ASC/β-actin, Cleaved-Caspase-1/β-actin and Gasdermin D-N/β-actin levels. **(B)** Representative TEM images of the proventriculus. Red arrows indicate mitochondrial damage; yellow arrows indicate cytoplasmic vacuolization. Scale bar = 2 μm. Data are presented as mean ± SEM (*n* = 3). *p < 0.05, **p < 0.01; ns, not significant.

Western blot analysis revealed that Cd exposure significantly upregulated expression of NLRP3, ASC, cleaved Caspase-1, and Gasdermin D-N in GS cells as compared to the Control group (Figure 4A), indicating activation of the canonical inflammasome-mediated pyroptotic pathway. Notably, AS-IV intervention markedly suppressed the protein levels of these pyroptosis-related markers, suggesting effective inhibition of Cd-induced pyroptotic signaling. TEM observation further validated these findings at the ultrastructural level (Figure 4B). The Control group exhibited intact plasma membranes and normal cellular morphology. In contrast, Cd-exposed GS cells displayed characteristic pyroptotic features, including plasma membrane swelling with the formation of numerous pores, cell lysis, and release of intracellular contents. Additionally, mitochondrial damage and cytoplasmic vacuolization were evident in the Cd group. Importantly, AS-IV treatment markedly ameliorated these ultrastructural abnormalities, as demonstrated by relatively intact plasma membrane integrity and preserved cellular architecture.

### 3.5 AS-IV alleviated Cd-induced necroptosis of duck GS cells

**Figure 5:**
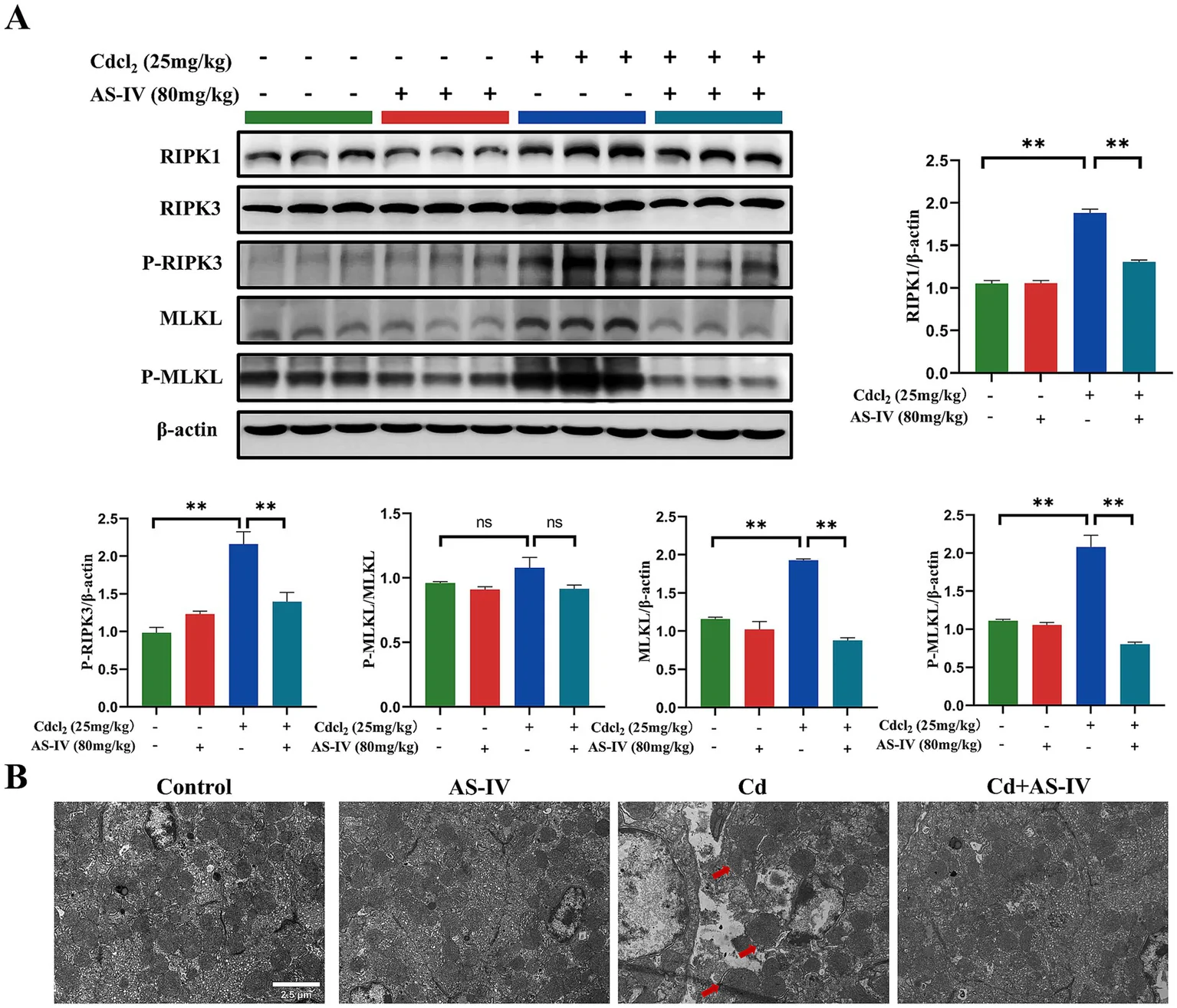
AS-IV alleviated Cd-induced necroptosis of duck GS cells. **(A)** Western blot results for RIPK1, RIPK3, P-RIPK3, MLKL, P-MLKL, and β-actin levels in proventriculus tissue; the ratios of RIPK1/β-actin, RIPK3/β-actin, P-RIPK3/β-actin, MLKL/β-actin, and P-MLKL/β-actin levels. **(B)** Representative TEM images of the proventriculus. Red arrows indicate mitochondrial damage. Scale bar = 2 μm. Data are presented as mean ± SEM (*n* = 3). *p < 0.05, **p < 0.01; ns, not significant.

WB analysis revealed that Cd exposure upregulated RIPK1 protein expression and markedly enhanced the levels of p-RIPK3 and p-MLKL (Figure 5A). Concurrently, total MLKL expression was modestly increased, whereas total RIPK3 remained relatively stable. These findings indicated activation of the canonical RIPK1-RIPK3-MLKL necroptotic signaling cascade. Notably, AS-IV intervention effectively suppressed RIPK1 upregulation and reduced p-RIPK3 and p-MLKL levels, suggesting inhibition of Cd-induced necroptotic kinase activation. TEM observation further corroborated these findings at the ultrastructural level (Figure 5B). The Control group exhibited intact plasma membranes, normal nuclear morphology, and well-preserved organelles. In contrast, Cd-exposed GS cells displayed characteristic necroptotic features, including plasma membrane rupture with loss of membrane integrity, organelle swelling, and release of cytoplasmic contents into the extracellular space. Additionally, mitochondrial dysfunction with disrupted cristae and nuclear membrane permeabilization without chromatin condensation were evident, distinguishing necroptosis from apoptotic morphology. Importantly, AS-IV treatment markedly ameliorated these ultrastructural abnormalities, with relatively preserved plasma membrane integrity and restored organelle morphology.

## 4 Discussion

Cd is increasingly released into the environment through industrial emissions, mining activities, and agricultural phosphate fertilizers, posing a significant threat to poultry health (21, 22). Field studies have documented that poultry raised in industrially contaminated areas exhibit significant Cd accumulation in multiple organs, resulting in necrotic lesions, tissue degeneration, and impaired production performance (23, 24). However, the adverse effects of Cd on the GS, a critical digestive and immune organ in poultry, have remained largely unexplored. In this study, we established a chronic Cd exposure model by supplementing the basal diet with CdCl₂ at 25 mg/kg for 28 consecutive days, which successfully induced marked glandular gastric injury as evidenced by significant enlargement of the GS, elevated GS index, severe histopathological alterations, including disrupted glandular tubular architecture, epithelial cell swelling, and vacuolar degeneration, along with basement membrane sloughing, mucosal necrosis, and prominent interstitial infiltration of inflammatory cells. These pronounced morphological and structural changes confirmed successful establishment of a duck model of Cd-induced glandular gastric injury, thereby providing a robust pathological foundation to investigate the protective effects and underlying mechanisms of AS-IV intervention.

The anti-inflammatory and antioxidant activities of AS-IV have been well characterized in diverse experimental disease models (25). In the present study, dietary supplementation with AS-IV (80 mg/kg BW) for 28 days markedly attenuated Cd-induced glandular gastric injury in ducks, as evidenced by a significant reduction in the GS index, amelioration of histopathological alterations, and restoration of Sirt3 protein expression. Oxidative stress and inflammatory responses are intricately linked pathological events that act synergistically to amplify tissue damage. Accordingly, bioactive natural compounds can confer tissue protection by simultaneously enhancing endogenous antioxidant defenses and suppressing deleterious inflammatory signaling pathways. The therapeutic potential of AS-IV is increasingly attributed to its dual antioxidant and anti-inflammatory capacity, which has been mechanistically associated with activation of the Nrf2 signaling cascade, consequent upregulation of downstream antioxidant enzymes, such as HO-1, and effective reduction of ROS accumulation in various experimental settings (26). Moreover, the protective efficacy of AS-IV against inflammation-driven tissue injury has been corroborated in multiple models. For instance, in cyclophosphamide-treated mice, AS-IV significantly alleviated oxidative stress and immunosuppression via the PI3K/Akt pathway (27). In septic models, AS-IV protected intestinal barrier integrity by suppressing signaling of the RhoA/NLRP3 inflammasome (28), collectively underscoring its pivotal regulatory role in oxidative-inflammatory networks. In line with these precedents, our biochemical analyses demonstrated that AS-IV pretreatment significantly elevated the GSH-Px content in the GS of Cd-exposed ducks. Notably, SOD activity and T-AOC also showed significant recovery, suggesting that AS-IV primarily mitigates Cd-induced oxidative insult by enhancing both the glutathione-dependent antioxidant system and the enzymatic antioxidant defense system rather than relying on a single pathway. Furthermore, AS-IV administered alone increased GSH-Px, SOD, and T-AOC levels, a pattern consistent with prior reports, indicating that AS-IV can enhance intrinsic antioxidant capacity and exert basal cytoprotective effects even in the absence of overt oxidative challenge (29). Mechanistically, the antioxidant and anti-inflammatory effects of AS-IV are functionally coupled. Cd-induced ROS overproduction activates redox-sensitive NF-κB and primes the NLRP3 inflammasome, driving IL-1β and IL-6 release. By restoring antioxidant enzyme activities, AS-IV quenches ROS accumulation, thereby suppressing downstream inflammatory signaling and disrupting the oxidative stress–inflammatory positive feedback loop to preserve gastric mucosal integrity. Consistent with this mechanistic framework, ELISA quantification in the current study revealed that AS-IV treatment significantly suppressed Cd-induced upregulation of the pro-inflammatory cytokines IL-1β and IL-6 in GS tissues, thereby confirming that AS-IV-mediated gastroprotection involves the concurrent attenuation of local inflammatory responses. Collectively, these results establish that AS-IV alleviates Cd-induced glandular gastric injury through a multi-pronged mechanism encompassing the enhancement of enzymatic and non-enzymatic antioxidant defenses and dampening of pro-inflammatory cytokine production, thereby preserving gastric mucosal integrity and function.

In Cd-sensitive organs, apoptosis, necroptosis, and pyroptosis do not occur in isolation, but rather are intricately interconnected through shared molecular nodes, synergistically amplifying tissue damage and inflammatory cascades, ultimately driving organ dysfunction. PANoptosis is orchestrated by the multi-protein PANoptosome complex, whereas dysregulation of this network triggers a vicious cycle of “cell death-inflammation,” exacerbating tissue pathological injury (30). Previous studies have demonstrated that AS-IV can exert organoprotective effects across various disease models by simultaneously targeting multiple pathways to inhibit both apoptosis and pyroptosis (31). For instance, in a model of cerebral ischemia–reperfusion injury, AS-IV significantly reduced neuronal apoptosis and parthanatos by stabilizing mitochondrial hexokinase-II, demonstrating its multifaceted intervention capacity against programmed cell death (32). Furthermore, in a model of oxygen–glucose deprivation/reoxygenation-induced neuronal injury, AS-IV effectively suppressed NLRP3 inflammasome-mediated pyroptosis and reduced Gasdermin D-N and IL-1β production through Nrf2 activation (33). However, previous investigations have predominantly focused on AS-IV-mediated regulation of individual cell death pathways, while it remains uncertain whether protective effects are conferred against heavy metal-induced gastrointestinal injury through coordinated modulation of the PANoptosis-related network. In this study, AS-IV pretreatment suggested broad-spectrum suppression of programmed cell death modalities. However, these findings represent indirect evidence and require functional validation (34). At the necroptotic level, AS-IV significantly inhibited p-MLKL membrane translocation and oligomerization, thereby blocking membrane integrity disruption and the subsequent inflammatory amplification mediated by DAMP release, consistent with the molecular characterization of RIPK family members as core regulatory nodes in PANoptosis (35). Concurrently, AS-IV treatment markedly suppressed NLRP3 inflammasome assembly and activation, downregulated expression of the related downstream effector molecules cleaved Caspase-1 and Gasdermin D-N, consequently reducing IL-1β maturation and release, while alleviating pore formation-driven cell lysis, which is consistent with a prior report that AS-IV inhibited podocyte pyroptosis via the TXNIP/NLRP3/Gasdermin D axis in diabetic nephropathy (36). Importantly, these three pathways are interconnected through shared molecular nodes, including RIPK1/caspase-8 signaling and the NLRP3 inflammasome. Consequently, ROS and DAMPs released during necroptosis and apoptosis can feedback to amplify pyroptotic signaling, establishing a self-sustaining PANoptotic network that AS-IV appears to disrupt at multiple convergent points. Taken together, AS-IV effectively mitigated Cd-induced injury in the duck GS by coordinately regulating PANoptosis-related pathways and simultaneously intervened in three modes of programmed cell death—apoptosis, necroptosis, and pyroptosis.

As a critical regulator of oxidative metabolism, mitochondrial homeostasis, and redox balance, and dysregulation of Sirt3, the principal NAD^+^-dependent mitochondrial deacetylase, has been implicated in the pathogenesis of various organ injuries (37). Recent evidence demonstrates that Sirt3 activation confers robust protection against tissue damage by suppressing mitochondrial ROS production, enhancing antioxidant enzyme activities, and modulating programmed cell death pathways (38, 39). For instance, in myocardial ischemia–reperfusion injury, Sirt3-mediated deacetylation of cyclophilin D limited mitochondrial permeability transition pore opening and attenuated necroptotic cell death (40). In the present study, Cd exposure significantly suppressed Sirt3 protein expression in the duck GS, whereas AS-IV pretreatment effectively restored Sirt3 expression to near control levels, suggesting that Sirt3 may serve as an upstream regulatory node in AS-IV-mediated gastroprotection. Based on these observations, we hypothesize that AS-IV may exert protective effects against Cd-triggered glandular gastric injury, at least in part, through the modulation of Sirt3 signaling. The resulting PPI network revealed that Sirt3 was associated with multiple molecules linked to PANoptosis, including RIPK1, NLRP3, and Caspase-3, suggesting possible regulatory crosstalk between mitochondrial redox regulation and inflammatory cell death pathways. Consistently, western blot analyses demonstrated that AS-IV pretreatment significantly inhibited Cd-induced alterations to the levels of molecules associated with the Sirt3 pathway, concomitant with the coordinated suppression of PANoptosis markers.

This study has several limitations. First, the PANoptosis framework adopted here integrates apoptosis, necroptosis, and pyroptosis based on coordinated molecular changes rather than direct PANoptosome assembly evidence. Second, conclusions rely primarily on gene and protein expression analyses, yielding associative rather than causal evidence. Third, while AS-IV pretreatment suppressed Sirt3 signaling and PANoptosis markers, our data support a regulatory association rather than confirmed upstream causality. As with prior natural product studies, validating direct mechanisms requires intervention approaches (e.g., pharmacological or genetic modulation). Accordingly, these findings should be viewed as reflecting coordinated pathway modulation. Future work employing Sirt3-specific agonists/antagonists or CRISPR-based editing is needed to establish causal links between Sirt3 activation and PANoptosis suppression in AS-IV-mediated protection against cadmium-induced gastric injury.

## 5 Conclusion

The present findings suggest that prophylactic AS-IV supplementation effectively attenuated cadmium-induced glandular gastric injury in ducks, evidenced by improved histopathology, normalized GS index, and reduced oxidative stress and inflammation. Mechanistically, these protective effects are associated with orchestrated regulation of PANoptosis-associated pathways, including concurrent suppression of apoptosis, necroptosis, and pyroptosis, potentially via Sirt3-mediated upstream signaling. These results not only offer a theoretical basis for employing botanical extracts to safeguard waterfowl gastrointestinal health against heavy metal toxicity, but also highlight the potential of AS-IV as a functional feed supplement for promoting health and sustainability in poultry production.

## Data Availability

The datasets presented in this study can be found in online repositories. The names of the repository/repositories and accession number(s) can be found at: https://doi.org/10.5281/zenodo.21150514.

## Footnotes

1
https://string-db.org/

## References

[1] Ye J Qiu W Pang X Su Y Zhang X Huang J . Polystyrene nanoplastics and cadmium co-exposure aggravated cardiomyocyte damage in mice by regulating PANoptosis pathway. Environ Pollut. (2024) 347:123713. doi: 10.1016/j.envpol.2024.123713, 38462200

[2] Lee H Jo Y Jung M Lee JH Kim TH Lee J . Heavy metal exposure and all health outcomes: an umbrella review of meta-analyses. J Hazard Mater. (2026) 503:141141. doi: 10.1016/j.jhazmat.2026.141141, 41564770

[3] Ma Y Su Q Yue C Zou H Zhu J Zhao H . The effect of oxidative stress-induced autophagy by cadmium exposure in kidney, liver, and bone damage, and neurotoxicity. Int J Mol Sci. (2022) 23:13491. doi: 10.3390/ijms232113491, 36362277 PMC9659299

[4] Wang X Mi J Yang K Wang L. Environmental cadmium exposure perturbs gut microbial Dysbiosis in ducks. Vet Sci. (2023) 10:649. doi: 10.3390/vetsci10110649, 37999472 PMC10674682

[5] Ma Y Su Q Zhao L Zhu J Zhao H Song R . Melatonin prevents cadmium-induced osteoporosis by affecting the osteoblast and osteoclast differentiation and pyroptosis in duck. Poult Sci. (2024) 103:103934. doi: 10.1016/j.psj.2024.103934, 38981361 PMC11294718

[6] Ma Y Ran D Zhao H Song R Zou H Gu J . Cadmium exposure triggers osteoporosis in duck via P2X7/PI3K/AKT-mediated osteoblast and osteoclast differentiation. Sci Total Environ. (2021) 750:141638. doi: 10.1016/j.scitotenv.2020.141638, 32858297

[7] Zou H Song J Luo X Ali W Li S Xiong L . Cadmium and polyvinyl chloride microplastics induce mitochondrial damage and apoptosis under oxidative stress in duck kidney. Poult Sci. (2025) 104:104490. doi: 10.1016/j.psj.2024.104490, 39571196 PMC11617461

[8] Zhang Q-L Qin W-X Li X-J Zhang Y-B Li M Xu J-F . Astragaloside IV is a potential natural neuroprotective agent for stroke: a review. Front Pharmacol. (2026) 16:1718700. doi: 10.3389/fphar.2025.1718700 41646941 PMC12868170

[9] Li Y Zhou J Zhang T Li X Wu C Zhao Z . Astragaloside IV attenuates cadmium induced nephrotoxicity in rats by activating Nrf2. Sci Rep. (2025) 15:2028. doi: 10.1038/s41598-025-86312-4, 39815001 PMC11735858

[10] Gong P Yue S Shi F Yang W Yao W Chen F . Protective effect of Astragaloside IV against cadmium-induced damage on mouse renal Podocytes (MPC5). Molecules. (2023) 28:4897. doi: 10.3390/molecules28134897, 37446560 PMC10343813

[11] Xi J Ma Y Liu D Li R. Astragaloside IV restrains pyroptosis and fibrotic development of pulmonary artery smooth muscle cells to ameliorate pulmonary artery hypertension through the PHD2/HIF1α signaling pathway. BMC Pulm Med. (2023) 23:386. doi: 10.1186/s12890-023-02660-9, 37828459 PMC10568875

[12] Tian H-Y Lei Y-X Zhou J-T Liu L-J Yang T Zhou Y . Insight into interplay between PANoptosis and autophagy: novel therapeutics in ischemic stroke. Front Mol Neurosci. (2025) 17:1482015. doi: 10.3389/fnmol.2024.1482015, 39846000 PMC11751022

[13] Zhang C Lin T Nie G Hu R Pi S Wei Z . Cadmium and molybdenum co-induce pyroptosis via ROS/PTEN/PI3K/AKT axis in duck renal tubular epithelial cells. Environ Pollut. (2021) 272:116403. doi: 10.1016/j.envpol.2020.116403, 33433347

[14] Zhang C Hu Z Hu R Pi S Wei Z Wang C . New insights into crosstalk between pyroptosis and autophagy co-induced by molybdenum and cadmium in duck renal tubular epithelial cells. J Hazard Mater. (2021) 416:126138. doi: 10.1016/j.jhazmat.2021.126138, 34492927

[15] Camilli S Madavarapu T El Ghissassi R Desaraju AB Busler C Soundararajan R . Determining the feasibility of a cadmium exposure model to activate the inflammatory arm of PANoptosis in murine monocytes. Int J Mol Sci. (2024) 25:10339. doi: 10.3390/ijms251910339, 39408668 PMC11476399

[16] Zai Z Qian X Xu Y Lv H Hao M Tao Y . ASIC1a induces excessive Mitophagy and PANoptosis of chondrocyte by the inhibition of SIRT3 mitochondrial translocation. Theranostics. (2025) 15:9623–42. doi: 10.7150/thno.116712, 41041065 PMC12486255

[17] Huang R Tang J Huang W Shang Y Liu W Long H . Compound Tinglizi decoction-containing serum is associated with inhibition of PANoptosis in Cardiomyocytes via SIRT3-related chaperone-mediated autophagy of AIM2. Int J Gen Med. (2025) 18:6975–87. doi: 10.2147/ijgm.S540452, 41262881 PMC12626036

[18] Hou L Yuan H Liu Y Sun X Chang J Zhang H . Effect of deoxynivalenol on inflammatory injury on the glandular stomach in chick embryos. Poult Sci. (2023) 102:102870. doi: 10.1016/j.psj.2023.102870, 37660451 PMC10491726

[19] Chen F Yang G Qiu H Gao S Hou L Dong J . Deoxynivalenol-induced pyroptosis and autophagy inhibition collectively promote inflammatory injury in the glandular stomach of chicken embryos. Poult Sci. (2025) 104:105052. doi: 10.1016/j.psj.2025.105052, 40120248 PMC11987648

[20] Yang Y Yao H He J Ou Z Huang Y Ba W . Betulinic acid attenuates lipopolysaccharide-induced kidney inflammatory injury by suppressing PANoptosis in weaned piglets. Vet Sci. (2026) 13:213. doi: 10.3390/vetsci13030213, 41893630 PMC13030141

[21] Duranova H Martiniakova M Omelka R Grosskopf B Bobonova I Toman R. Changes in compact bone microstructure of rats subchronically exposed to cadmium. Acta Vet Scand. (2014) 56:64. doi: 10.1186/s13028-014-0064-0, 25279860 PMC4189194

[22] Wang L Cui X Cheng H Chen F Wang J Zhao X . A review of soil cadmium contamination in China including a health risk assessment. Environ Sci Pollut Res Int. (2015) 22:16441–52. doi: 10.1007/s11356-015-5273-1, 26362640

[23] Elkribi-Boukhris S Boughattas I Sappin-Didier V Helaoui S Coriou C Bussiere S . Exposure to polymetallic contaminated sites induced toxicological effects on chicken lungs: a multi-level analysis. Chemosphere. (2024) 354:141574. doi: 10.1016/j.chemosphere.2024.141574, 38460845

[24] Kar I Mukhopadhayay SK Patra AK Pradhan S. Bioaccumulation of selected heavy metals and histopathological and hematobiochemical alterations in backyard chickens reared in an industrial area, India. Environ Sci Pollut Res. (2018) 25:3905–12. doi: 10.1007/s11356-017-0799-z 29177781

[25] Yang J Xu H Ye R Zhang Y Wu J Jin E . Astragaloside IV: a natural shield against ochratoxin A-induced hepatotoxicity in chicks by targeting the NRF2/NLRP3 signaling pathway. Toxicon. (2025) 266:108513. doi: 10.1016/j.toxicon.2025.108513, 40780305

[26] Su X Guo H Zhou Y Cao A Shen Q Zhu B . Astragaloside IV attenuates high glucose-induced NF-κB-mediated inflammation through activation of PI3K/AKT-ERK-dependent Nrf2/ARE signaling pathway in glomerular mesangial cells. Phytother Res. (2023) 37:4133–48. doi: 10.1002/ptr.7875, 37189016

[27] Sun Y Cao J Li M Ma J Wang W. Astragaloside IV improves cyclophosphamide-induced liver injury in mice by mediating immunosuppression and oxidative stress through the PI3K/Akt signaling pathway: a randomized trial. Hepat Mon. (2025) 25:e164840. doi: 10.5812/hepatmon-164840

[28] Xie S Yang T Wang Z Li M Ding L Hu X . Astragaloside IV attenuates sepsis-induced intestinal barrier dysfunction via suppressing RhoA/NLRP3 inflammasome signaling. Int Immunopharmacol. (2020) 78:106066. doi: 10.1016/j.intimp.2019.106066, 31835087

[29] Shao A Guo S Tu S Ammar AB Tang J Hong Y . Astragaloside IV alleviates early brain injury following experimental subarachnoid hemorrhage in rats. Int J Med Sci. (2014) 11:1073–81. doi: 10.7150/ijms.9282, 25136262 PMC4135229

[30] Wang Y Kanneganti TD. From pyroptosis, apoptosis and necroptosis to PANoptosis: a mechanistic compendium of programmed cell death pathways. Comput Struct Biotechnol J. (2021) 19:4641–57. doi: 10.1016/j.csbj.2021.07.038, 34504660 PMC8405902

[31] Shi N Wei L Wang H Sun S Yang J Zhou Y. Regulation of pyroptosis by natural products in atherosclerosis: mechanisms and therapeutic potential. Front Pharmacol. (2025) 16:1626566. doi: 10.3389/fphar.2025.1626566 40978461 PMC12443756

[32] Li Y Yang Y Zhao Y Zhang J Liu B Jiao S . Astragaloside IV reduces neuronal apoptosis and parthanatos in ischemic injury by preserving mitochondrial hexokinase-II. Free Radic Biol Med. (2019) 131:251–63. doi: 10.1016/j.freeradbiomed.2018.11.033, 30502455

[33] Xiao L Dai Z Tang W Liu C Tang B. Astragaloside IV alleviates cerebral ischemia-reperfusion injury through NLRP3 inflammasome-mediated pyroptosis inhibition via activating Nrf2. Oxidative Med Cell Longev. (2021) 2021:9925561. doi: 10.1155/2021/9925561, 35003524 PMC8739174

[34] Hou B Liu R Wu Y Huang S. Astragaloside IV reduces cerebral ischemia/reperfusion-induced blood-brain barrier permeability in rats by inhibiting ER stress-mediated apoptosis. Evid Based Complement Alternat Med. (2020) 2020:9087873. doi: 10.1155/2020/9087873, 33193803 PMC7641265

[35] Gao J Xiong A Liu J Li X Wang J Zhang L . PANoptosis: bridging apoptosis, pyroptosis, and necroptosis in cancer progression and treatment. Cancer Gene Ther. (2024) 31:970–83. doi: 10.1038/s41417-024-00765-9, 38553639 PMC11257964

[36] Hu Z Zhou Y Gao C Liu J Pan C Guo J. Astragaloside IV attenuates podocyte apoptosis via regulating TXNIP/NLRP3/GSDMD signaling pathway in diabetic nephropathy. Diabetol Metab Syndr. (2024) 16:296. doi: 10.1186/s13098-024-01546-y, 39696607 PMC11656976

[37] Tran QA Tran GV Velic S Xiong HM Kaur J Moosavi Z . Effects of Astragaloside IV and Formononetin on oxidative stress and mitochondrial biogenesis in hepatocytes. Int J Mol Sci. (2025) 26:774. doi: 10.3390/ijms26020774, 39859490 PMC11765978

[38] Yapryntseva MA Maximchik PV Zhivotovsky B Gogvadze V. Mitochondrial sirtuin 3 and various cell death modalities. Front Cell Dev Biol. (2022) 10:947357. doi: 10.3389/fcell.2022.947357, 35938164 PMC9354933

[39] Ilari S Giancotti LA Lauro F Gliozzi M Malafoglia V Palma E . Natural antioxidant control of neuropathic pain-exploring the role of mitochondrial SIRT3 pathway. Antioxidants (Basel). (2020) 9:1103. doi: 10.3390/antiox9111103, 33182469 PMC7698145

[40] Du B Fu Q Yang Q Yang Y Li R Yang X . Different types of cell death and their interactions in myocardial ischemia-reperfusion injury. Cell Death Discov. (2025) 11:87. doi: 10.1038/s41420-025-02372-5, 40044643 PMC11883039

